# Medicine

**Published:** 1904-06

**Authors:** G. Parker


### Progress of the Medical Sciences.

### MEDICINE.

**Complications in Typhoid.** A comparatively unrecognised danger has been discussed by Thayer[1] who, by examining the condition of a hundred and eighty-two persons who had previously suffered from typhoid, shows that the disease has some **tendency to produce arterio-sclerosis.** Thus he found that the average systolic blood pressure was higher in them than in a number of normal individuals. This was constant in every decade of life, and in many instances the rise was markedly abnormal. The radial arteries could be palpated nearly three times as often as in healthy persons, and the size of the heart was on the average increased since the illness. Cardiac murmurs were frequent, eight cases of mitral insufficiency alone being noted among those whose hearts were normal during the attack, while those in whom a systolic apex murmur was noted during the illness showed a marked increase of blood pressure and of the size of the heart. The writer concludes that these post typhoid cases were materially aged by the disease as regards the heart and arteries, and that it is a probable cause of a fair number of cases of hypertrophy and dilatation in early and middle life. Landouzy had previously noticed cardiac changes in a few old cases of typhoid, and it will be important to see whether other observers corroborate these results. Possibly, as Manges says, all the acute infectious diseases tend to cause these arterio-sclerotic changes; but, after all, when we consider the frequency with which degenerative changes in the heart muscle are known to occur during typhoid, the results of Thayer's investigation into the after-state of convalescents are not astonishing.

To turn to another group of sequelæ, the nervous affections. "There is scarcely any other febrile affection, except perhaps influenza, which is followed by so many nervous sequelæ as typhoid" (Dreschfeld). Still, these are usually temporary, though they predispose the patient to functional disorders such as hysteria and melancholia. However, disseminated sclerosis, myelitis, hemiplegia, and even bulbar paralysis have been recorded. Neuritis, especially of the ulnar and peroneal nerves, ptosis,

[1] *Am. J. M. Sc.*, 1904, cxxvii. 391.

and affections of the recurrent laryngeal are not very rare, and the typhoid spine is possibly due to nerve lesions.[1]

**Rigors** may be considered as belonging to the same group. They are rare, the cause is in most cases unknown, and they do not greatly affect the prognosis, except when due to some definite complication. Ch. Bolton[2] analyses the literature of the subject, and describes a case where twenty-one rigors occurred. The first one came on with a hemorrhage during diarrhœa ; later ones were without any complication. He takes the view that some irritation of the intestinal mucous membrane is the exciting cause in many instances. Even undressing, sponging, an enema, or the passage of a motion may suffice when the thermotaxic centre has been rendered unstable by the poison of the disease. Thus Herringham points out that they are commoner during lysis. Bouveret noted the same fact, though he would look on them as partly due to the sudden absorption of a large dose of toxin. Church recorded fifty-three rigors in the course of one case which ended favourably, while Osler notes their occasional occurrence at the onset of the disease or of a relapse. In a number of secondary cases the rigors are due to complications such as thrombosis of the mesenteric veins, small abscesses and septic absorption, and even perforation itself. Hence if a rigor occurs the first thing is to decide whether it is due to one of these definite complications, or is merely the symptom of an unstable nerve state under the strain of the illness.

**Perforation.**—According to the figures quoted by Hector Mackenzie,[3] out of a hundred cases treated in the London hospitals there are 13.4 deaths, and under the Metropolitan Asylums Board 15.6, while under the Brand bathing system, thoroughly carried out as in some continental countries and at Montreal, at Brisbane and at the Johns Hopkins Hospital there are only about seven deaths, that is, seven lives in every hundred sick are saved. If the mortality in the rest of England and Wales is as high as in London this would amount to the annual loss of some 3,000 lives owing to difficulties in carrying out the best plan of treatment. If any advocates of ice cradles, wet packs or affusion, could show statistics approaching these they would be eagerly welcomed. The reduction of mortality, however, is chiefly due to the elimination of toxines, and to the fewer deaths from toxæmia. The complications, such as perforation, are hardly affected. Thus the mortality in the London hospitals from perforation is 4.5 per hundred, and very little less abroad. There are some 1,500 deaths from it every year in England and Wales. Have we any means of preventing them ?

All authorities agree that at present our only remedy lies in early diagnosis and immediate surgical treatment. If we wait till general peritonitis has set in it is usually too late to

[1] Pearce Bailey, *Med. Rec.*, 1903, lxiv. 835.

[2] *Practitioner*, 1904 lxxii. 106. [3] *Lancet*, 1903, ii. 863.

do any good. The complication rarely occurs before the tenth day, most often it is seen during the third week or at the beginning of the fourth, or in a relapse about the end of the second week. Now the symptoms are most varied and sometimes indefinite, so that physicians and nurses need to be constantly on the look-out for them. If early recognition and efficient surgical treatment were the rule it is estimated that 400 or 500 lives could be saved annually. Harte and Ashhurst,[1] in commenting upon 364 cases thus treated, point out that suspicion should attach at once to stabbing or sudden pain in the abdomen, especially in apathetic patients who have some distension of the bowels or loss of control of the sphincters. This pain may vary enormously in amount, and may be situated in the lower and especially in the right side of the abdomen, or may be referred to the bladder or even to the end of the penis. Vomiting, nausea, hiccough, diarrhœa, rigors, a sudden rise or fall of temperature, a marked change in the facial expression, or an increase in the pulse or respiration rate, may any of them accompany the pain. An area of abdominal tenderness, most often in the right lower quadrant, may be suddenly developed. Still more important is any rigidity of the recti muscles, especially of the right one, when not produced by careless palpation. Softness of the abdomen and free respiratory movements, and the absence alike of distension and retraction, are favourable signs. The sudden development again of dulness in the flanks shifting with the patient's position must never be neglected. All these points should be considered before the arrival of the stage of general peritonitis with the board-like abdomen and general septic state. Any one of the symptoms may be absent, almost any one of them may be due to other causes. We must weigh and collect all the possible evidence, noting such things as the drawing up of the legs, or a running, thready pulse. The liver dulness may be examined, but little reliance can be placed upon it. The blood count is not yet an exact index of the condition, though a steadily increasing leucocytosis made up chiefly from polynuclear cells is not to be overlooked, but it may occur also from phlebitis, cholecystitis, and other complications. A recent suggestion by J. B. Briggs, of Johns Hopkins, may, if confirmed, prove to be a valuable sign. He claims that perforation is accompanied with a sudden rise of blood pressure as shown by the sphygmomanometer.[2] Cushing had previously noted a sharp rise in blood pressure when the peritoneum is irritated during abdominal operations. Briggs took observations on some typhoid cases, and in one where perforation actually occurred found that the pressure suddenly rose from 106 to 144 mm. before any other symptoms appeared, while in another where some signs of perforation were present and laparatomy was performed, but no perforation was found, there was no rise. It is not likely that any ordinary complication could thus affect the blood pressure,

[1] *Ann. Surg.*, 1904, xxxix. 8. [2] *Boston M. and S. J.*, 1903, cxlix. 343.

and further confirmation of the theory will be eagerly awaited as affording one reliable and early sign.

**Ankylostomiasis.** Human parasites seem to find little difficulty in accompanying their hosts to this climate whatever are the difficulties in acclimatising the higher plants and animals. At the present time, owing to the facilities of travel, we are threatened with hosts of new companions. Our old invaders, the bug and the rat, are being followed, not only by the bacilli of plague and cholera, but by the bilharzia, the filaria, the nematode of "craw craw," the ankylostoma, and others. We may disregard the trypanosoma in our midst if the infection cannot be spread, but it is now said that the infection of bilharzia is liable to conveyance by the urine or fæces. In the neighbourhood of Birmingham the West Indian "craw craw" has gained a footing, and patients are there found tormented with an intolerable itching which is succeeded by crops of vesicles on the trunk and limbs.[1] The disease has been brought by travellers returning from the tropics, while soldiers from the Rustenberg district in South Africa are largely responsible for the bilharzia. Far more serious than these, especially in a mining district like ours, is the invasion by the ankylostoma, which threatens to become a veritable scourge. Before long, if the disease is not checked, extreme anæmia will be more common among male out-patients than the chlorosis of women. The original habitat of the worm lay in districts not more than 35° from the equator, but since its discovery in the labourers at the St. Gothard tunnel it has spread over the southern states of America, over Hungary, Germany, Belgium, France, and now it is rife in Cornwall, and has even been found in Scotland. The adult worms live in the upper part of the small intestine, each worm having a life span of some years, and the females produce an enormous stream of eggs which hatch at a temperature of 60° and upwards, and are conveyed in the larval form to new hosts by contaminated food and water. Boycott and Haldane,[2] in describing recent investigations upon the life history of this parasite, show reason to believe that the infection can also be conveyed through the skin, which increases very largely the difficulties of dealing with it. Bentley found that the water-itch, which attacks the bare feet of coolies in the Assam tea gardens, can be produced by applying to the skin moist earth mixed with the ova of the ankylostoma, and the worm has been found in the intestine of the patient after such treatment. Thus it would appear that water-itch itself is but one of the results of the parasite. Among the Cornish miners who are infected, though the feet are protected by boots, troublesome skin eruptions called "bunches" are common and are probably due to the same cause. Nearly all the men in certain mines are attacked, though some become immune and show few symptoms while still harbouring the parasite. Others develop

[1] Walker Hall, *Med. Chron.*, 1904, 4 s., vii. 2. [2] *J. Hygiene*, 1904, iv. 73.

extreme anæmia, breathlessness, and gastro-intestinal troubles with eosin changes in the blood, with sometimes a fatal termination. In some Westphalian collieries 17,000 men were found to be infected, while in certain tropical countries nearly the whole population are attacked, to the great increase of the mortality and to the serious lessening of the efficiency of the labourer. In colder countries there is a slight natural protection, for it is only in warm mines that the eggs can develop into larvæ, and it is chiefly as larvæ that they can most easily get an entrance into the body of a new host. Still, even in the neighbourhood of Cologne, the brickfields have proved to be nurseries of the disease. In warm summers in this country the eggs might hatch in the open air, but the great danger will always be in the mines, and especially damp ones. Thus an outburst of the disease occurred in certain Westphalian mines, where the dusty roads were watered to prevent explosions of gas. The discovery of eggs in the fæces is easy if watery films are placed under the microscope, when they are seen as oval, smooth bodies, about 59 by 37$\mu$ in size, with a pellucid shell bounded by a single line, and grey contents. The worms themselves are whitish, and about half an inch long, with two pairs of pointed hooks on the ventral surface of the mouth. They are found in the fæces after a dose of thymol if the bowels act quickly; otherwise they are broken up and digested by the pancreatic juice soon after they are killed by the thymol. In Cornwall the treatment consists of a preliminary purge and then three doses of thymol of thirty grains each at intervals of two hours, and finally another aperient, alcohol and castor oil being carefully avoided to prevent the risk of poisoning from their combination with thymol. These doses must be repeated at intervals till the stools are free from eggs. In Westphalia large doses of male fern are given more than once, but the risks of collapse and even blindness are somewhat serious. In short, any effective treatment is difficult and unpleasant, and though by vigorous measures the disease in Westphalia has been greatly reduced, prevention is all important. To stop the pollution of the mines by the fæces of infected persons, to provide underground privies properly disinfected, and to warn the men of the danger of eating with hands soiled by the earth are among the most important safeguards. It may be necessary to allow no new hands to begin work until their fæces have been tested, especially if they come from infected mines. Boycott and Haldane think that the good ventilation of most English coal mines reduces the risk of infection by drying the ground and lowering the temperature, but even this cannot always be relied upon. In short, we are threatened by a very serious danger, and the means of meeting it do not appear so complete and so effective as we could wish. Hence the necessity of recognising, treating, and isolating every case as early as possible.

G. PARKER.

